# Early acquisition and high nasopharyngeal co-colonisation by *Streptococcus pneumoniae *and three respiratory pathogens amongst Gambian new-borns and infants

**DOI:** 10.1186/1471-2334-11-175

**Published:** 2011-06-20

**Authors:** Brenda A Kwambana, Michael R Barer, Christian Bottomley, Richard A Adegbola, Martin Antonio

**Affiliations:** 1 Bacterial Diseases Programme, Medical Research Council Laboratories (UK), Atlantic Boulevard, Banjul, P. O. Box 273, The Gambia; 2 Department of Infection, Immunity, and Inflammation, Maurice Shock Medical Sciences Building, University Road, Leicester, LE1 9HN, UK; 3 Infectious Disease Epidemiology Unit, London School of Hygiene and Tropical Medicine, Keppel Street, London, WC1E 7HT, UK; 4 Infectious Diseases Development, Global Health Program, Bill & Melinda Gates Foundation, Seattle, WA 98102, USA

**Keywords:** Nasopharyngeal, PCR, respiratory pathogens

## Abstract

**Background:**

Although *Haemophilus influenzae type b *(Hib), *Staphylococcus aureus *and *Moraxella catarrhalis *are important causes of invasive and mucosal bacterial disease among children, co-carriage with *Streptococcus pneumoniae *during infancy has not been determined in West Africa.

**Methods:**

Species specific PCR was applied to detect each microbe using purified genomic DNA from 498 nasopharyngeal (NP) swabs collected from 30 Gambian neonates every two weeks from 0 to 6 months and bi-monthly up to 12 months.

**Results:**

All infants carried *S. pneumoniae, H. influenzae *and *M. catarrhalis *at several time points during infancy. S.*pneumoniae *co-colonized the infant nasopharynx with at least one other pathogen nine out of ten times. There was early colonization of the newborns and neonates, the average times to first detection were 5, 7, 3 and 14 weeks for *S. pneumoniae, H. influenzae, M. catarrhalis *and *S. aureus *respectively. The prevalence of *S. pneumoniae, H. influenzae *and *M. catarrhalis *increased among the neonates and exceeded 80% by 13, 15 and 23 weeks respectively. In contrast, the prevalence of *S. aureus *decreased from 50% among the newborns to 20% amongst nine-week old neonates. *S. pneumoniae *appeared to have a strong positive association with *H. influenzae *(OR 5.03; 95% CI 3.02, 8.39; p < 0.01) *and M. catarrhalis *(OR 2.20; 95% CI 1.29; p < 0.01) but it was negatively associated with *S. aureus *(OR 0.53; 95% CI 0.30, 0.94; p = 0.03).

**Conclusion:**

This study shows early acquisition and high co-carriage of three important respiratory pathogens with *S. pneumoniae *in the nasopharyngeal mucosa among Gambian neonates and infants. This has important potential implications for the aetiology of respiratory polymicrobial infections, biofilm formation and vaccine strategies.

## Background

An astounding 83% of childhood deaths (< 5 years) between 1970 and 2009 occurred in Asia and sub-Saharan Africa, sharply contrasted with less than 1% which occurred in high-income nations [1]. Invasive bacterial disease (IBD) such as pneumonia, meningitis and bacteraemia contribute to the disparity in childhood mortality in developing and developed countries [2,3]. 18% of the estimated eight million childhood deaths (< 5 years) which occurred worldwide in 2008 were attributed to pneumonia, making it the single commonest cause of death in the under five year olds [4].

Approximately 50% of the severe pneumonia cases in developing countries are attributed to *Streptococcus pneumoniae *and *Haemophilus influenzae type b *(Hib) in areas where the vaccine is not widely available [5]. Non-typeable strains of *H. influenzae *(NtHi) are associated with otitis media (OM), community acquired pneumonia and other IBD among vulnerable populations [6-8]. OM is the most common bacterial infectious disease amongst children [9] and up to 20% of acute OM episodes are caused by *Moraxella catarrhalis *[10]. In the Gambia, pneumococcal nasopharyngeal carriage occurs rapidly after birth and carriage exceeds 80% in infants [11]. Not surprisingly, *S. pneumoniae *is also the leading cause of IBD [12], with the highest prevalence occurring in infancy. *S. pneumoniae *and *Staphylococcus aureus *accounted for 45.2% and 18.3% respectively of bacteraemia cases in a study amongst hospitalised patients in the Gambia with a median age of 2 years (range 2 months to 80 years) [13].

The nasopharynx is an important reservoir of commensal and pathogenic microbes which can migrate to the sinuses, middle ear and lower respiratory tract and invade the blood system. Nasopharyngeal carriage is thought to be the main source of transmission of pathogens across individuals [14,15]. Although the specific mechanisms are poorly understood, attachment to the nasopharyngeal epithelial surface is thought to be an essential step in the development of mucosal and invasive disease [16,17]. The epidemiology, transmission and nasopharyngeal carriage of *S. pneumoniae *have been studied in the Gambia [11,13,15,18]. However co-carriage of *S. pneumoniae *with *H. influenzae, S. aureus *and *M. catarrhalis *has not been described in West Africa.

The seven-valent pneumococcal polysaccharide-diphtheria CRM_197 _protein conjugate vaccine (PCV-7) markedly reduces the carriage of vaccine serotypes and decreases the incidence of vaccine serotype invasive disease, making it a remarkable public health success story [19]. However, the long term effectiveness of PCV-7 also depends on the emergence of serotype and species replacement disease as the vacant niche is replenished by non-vaccine pneumococcal serotypes or possibly other respiratory pathogens that share the nasopharyngeal niche [20,21].

This study set out to determine the co-carriage of *H. influenzae, S. aureus *and *M. catarrhalis *with *S. pneumoniae *in the nasopharynx amongst PCV-7 naïve infants using PCR-based methods.

## Methods

### Study site and sample collection

Nasopharyngeal (NP) swabs were collected from infants from the Western Region of the Gambia after obtaining parental informed consent as previously described by Hill et *al*., 2008 [11]. Approval to conduct this study was sought from the MRC, The Gambia Scientific Coordinating Committee and the Joint MRC & Gambian Government Ethics Committee. NP swabs were collected prior to the introduction of PCV-7. NP swabs were collected by using sterile calcium alginate fibre tipped swabs with aluminium shafts (Fisher Brand^® ^, USA), placed in vials containing Skim milk-tryptone-glucose-glycerol (STGG) transport medium and stored at -70°C up to the time this study was conducted. Thirty infants from which 498 NP swabs (n = 16 NP swabs/infant) were selected from a larger cohort of 236 subjects on the basis that they had complete or nearly complete data sets collected according to the study design as described in [11]. The expected number of NP swabs per infant was 17 however; the average number of NP swabs amongst the infant selected was 16.6. Each subject had the first NP swab collected within a week of birth, bi-weekly for the first 6 months and then bi-monthly for another 6 months.

### DNA isolation

The NP swabs in STGG and stored at -70°C were thawed at room temperature (25°C) and vortexed for approximately 10-20 s. 100 μL were centrifuged at *5 000 × g *for 10 minutes and the pellet was resuspended in 20 mg/mL lysozyme in lysis buffer (20 mM Tris-Cl, pH 8.0, 2 mM sodium EDTA, 1.2% Triton^® ^X-100). DNA was extracted using the DNeasy Blood & Tissue Kit^® ^(QIAGEN, UK), following the manufacturer's Gram positive protocol. DNA was eluted in 100 μL of elution buffer and stored at -20°C. Extractions were done in batches of 24 including a negative control to which DNAse/RNase free water was added and a positive control to which a fresh *S. pneumoniae *cell suspension (1 × 10^4 ^cells/mL) was added.

### PCR detection of pathogens

PCR reactions were carried out in 25 μL reaction volumes consisting 2.5 μL (10 ng) of purified genomic DNA, 1} Green GoTaq^® ^reaction buffer with 1.5 mM MgCl_2_, 1.0 U Go*Taq *Polymerase (Promega, UK), each deoxynucleoside triphosphate at 0.2 mM (dNTP) (QIAGEN, UK). The gene targets and primer information specific for each microbe are described below. PCR controls were purified genomic DNA (20 ng/μL) of the following clinical isolates; *S. pneumoniae, S. aureus*, Hib, NtHi, *M. catarrhalis, Pseudomonas aeruginosa, Escherichia coli*, Group A *Streptococcu*s, Group B *Streptococcus, N. meningitidis, Citrobacter freundii, Shigella flexneri *and coagulase negative *Staphylococcus*. Thermal cycling was performed in the Gradient Palm-Cycler™ (Corbett Life Sciences, UK) as follows, 95°C for 2 minutes, followed by 35 cycles of 95°C for 30 seconds, 60°C for 30 seconds and 72°C for 45 seconds and a final extension of 72°C for 10 minutes.

Detection of *S. pneumoniae *was carried out using primers that target the capsular polysaccharide synthesis gene locus A, (*cpsA*), *cpsA*f:5'-GCAGTACAGCAGTTTGTTGGACTGACC-3' and *cpsA*r:5'-GAATATTTTCATTATCAGTCCCAGTC-3' [22]. *M. catarrhalis *detection was done using primers that target the outer membrane protein *copB *gene, copBf:5'-GTGAGTGCCGCTTTTACAACC-3' and copBr:5'-TGTATCGCCTGCCAAGACAA-3' [23]. *S. aureus *was detected using primers that target the Thermonuclease (Tnase) encoding gene (*nuc*), nucf:5'-GCGATTGATGGTGATACGGTT-3'5 and nucr:5'-AGCCAAGCCTTGACGAACTAAAGC-3' [24]. *H. influenzae *detection was done using primers that target the Outer membrane lipoprotein P2 (*OmpP2)*, ompP2f:5'-GGTGCATTCGCAGCTTCAG-3' and ompP2r:5'GATTGCGTAATGCACCGTGTT-3'[25]. Capsule detection and typing of Hib were carried using the oligonucleotides described by Howie *et al*. [26].

16S PCR detection was carried out on NP swabs that did not yield any of the pathogens assayed using the following primers: 338f-5'-ACTCCTACGGGNGGCNGCA-3' and 1046r-5'-CACGAGCTGACGACANCCATGCANCACC-3'. The presence of PCR inhibitors was determined by spiking 20 μL of the NP swab genomic DNA with 10 ng of *S. pneumoniae *DNA. 16S rRNA gene PCR was conducted as described above on samples that were negative for all four pathogens. 5 μL of PCR product were loaded on 2% agarose gels stained with ethidium bromide and analyzed by gel electrophoresis in 1x TAE buffer (40 mM Tris, 20 mM of glacial acetic acid, 1 mM EDTA, pH 8.0) for 60 min at 100 V. Gel images were recorded and the sizes of the PCR products were confirmed by comparison with the molecular size standard HyperLadder II (Bioline, UK).

### Statistical Analysis

Results of the organism specific PCR assays were recorded as presence/absence outcomes and all the statistical analyses were carried out in STATA release 11 (StataCorp LP, USA). The point prevalence of each pathogen was determined for the 16 age strata ranging from (1, 3, 5, 7, 9 weeks up to 27 weeks and then 35, 43 and 51 weeks). Logistic regression was used to determine associations between carriage of S. pneumoniae and the presence of each of the other three pathogens. Confounding factors in the adjusted model included age, ethnic group, sex, antibiotic treatment and type of feeding [27]. Sixteen to seventeen samples were collected per infant; hence, to account for correlations of response variables from the same infant, subject identity was entered as a random effect. Log likelihood Ratio Tests were performed to determine the contribution of each pathogen to the model. Kaplan-Meier survivor function was used to conducted time to first detection for each of the microbes.

## Results

Species specific PCR was applied to detect four pathogens in 498 NP swabs collected from 30 children in the first year of life (n = 16.6NP swabs/infant). At least one pathogen was detected in 473 (95%) of the nasopharyngeal swabs. The presence of bacteria was confirmed by 16S rRNA gene PCR for 15 (3%) of the nasopharyngeal swabs that were negative for all four pathogens. Amplification of spiked NP swab DNA contraindicated the presence of inhibitors in the 5 (2%) NP swabs which were negative for any of the pathogens and the 16S rRNA gene.

### High co-carriage of *S. pneumoniae *and respiratory pathogens

**Figure 1:**
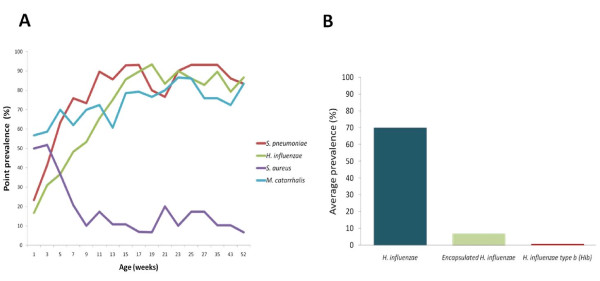
**Carriage of *S. pneumoniae *and three respiratory pathogens in the first year of life**. The point prevalence of *S. pneumoniae *and three respiratory in the nasopharynx among 30 infants followed-up from birth to one year (A). The average prevalence of *H. influenzae*, encapsulated *H. influenzae *and Hib in the nasopharynx among 30 infants followed-up from birth to one year (B).

Of the 391 NP swabs in which *S. pneumoniae *was found, it was the only pathogen detected in 25 (6%) and it was detected with at least one other pathogen in 366 NP swabs (94%). *S. pneumoniae *was found with one, two and three pathogens in 102 (26%), 228 (58%) and 36 (9%) 391 NP swabs respectively. During infancy, the average carriage prevalence for each of the pathogens was as follows; *S. pneumoniae *78% (95CI: 76%, 83%), *M. catarrhalis *71% (95CI: 67%, 75%), *H. influenzae *70% (95CI: 65%, 74%) and *S. aureus *20% (95CI: 16%, 24%). Carriage of both *S. pneumoniae *and *H. influenzae *was below 30% in the first week but increased rapidly as the infants got older, exceeding 90% between 15 and 19 weeks respectively (Figure 1). *M. catarrhalis *carriage was 57% in the first week and increased gradually to above 80% by 21 weeks. In contrast, *S. aureus *carriage was 50% in the first week and rapidly fell to 10% by 9 weeks and remained low thereafter (Figure 1). The average prevalence of encapsulated *H. influenzae *was 7% (95CI: 4.6%, 9.2%) and Hib had average prevalence of 0.7% (95CI: 0.5%, 0.9%) in the NP swabs assayed (Figure 1).

### Early acquisition of *S. pneumoniae *and respiratory pathogens

**Figure 2:**
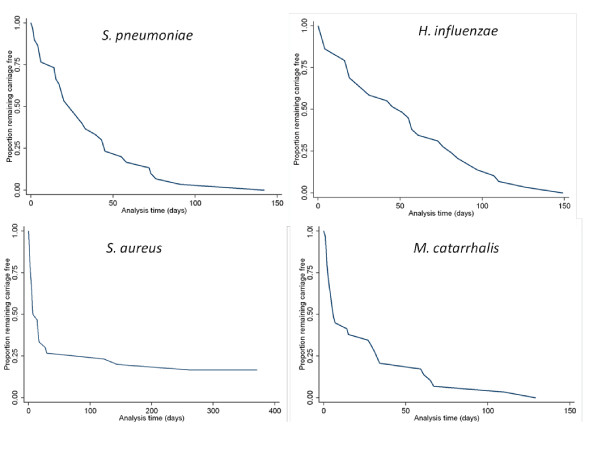
**Survival curve for time to first detection of *S. pneumoniae *and three respiratory pathogens among 30 infants followed-up from birth to one year**.

**Figure 3:**
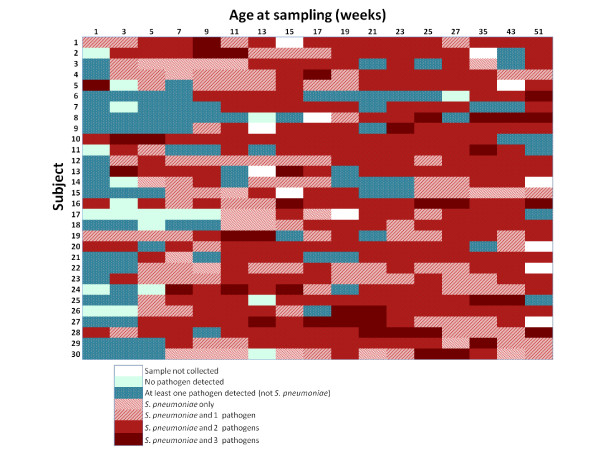
**Heatmap showing co-carriage of *S. pneumoniae *with three respiratory pathogens in the nasopharynx among 30 infants followed up from birth to one year**.

The average times to first detection of *S. pneumoniae, H. influenzae, M. catarrhalis *and *S. aureus *were 5, 7, 3 and 14 weeks respectively. By 129 days, 142 days and 149 days all the infants had carried *M. catarrhalis, S. pneumoniae *and *H. influenzae *at least once respectively (Figure 2). Within the first 30 days, more than 75% of the infants had acquired *S. aureus *at least once, (Figure 2); however, this pathogen was not detected in any of the NP swabs from 5 (17%) of the infants. 25 (83%) of the samples collected soon after birth (< 6 days) had at least one pathogen detected, see Figure 3.

### Associations between *S. pneumoniae *and three respiratory pathogens

Logistic regression modelling was used to determine the univariate and adjusted associations between *S. pneumoniae *carriage and the other respiratory pathogens. The adjusted model included the following confounding factors; age, ethnic group, sex, antibiotic treatment, type of feeding and weight [11,27]. There was a significant positive interaction between colonization with *S. pneumoniae *and *H. influenzae *in univariate analysis (OR 5.03; 95% CI 3.02, 8.39; p < 0.01) and the adjusted analysis OR 2.02; 95% CI 1.04, 3.91; p = 0.04). A significant positive association was found between *S. pneumoniae *and *M. catarrhalis *in univariate analysis (OR 2.20; 95% CI 1.29; p < 0.01) but not in the adjusted analysis (OR 1.40; 95% CI 0.72, 2.71; p = 0.33). A significant negative correlation was found between *S. pneumoniae *and *S. aureus*, (OR 0.53; 95% CI 0.30, 0.94; p = 0.03) but not after the adjusted analysis (OR 1.33; 95% CI 0.59, 3.02; p = 033).

## Discussion

For the first time in West Africa, the nasopharyngeal carriage and co-occurrence of *S. pneumoniae *with respiratory pathogens, *H. influenzae, S. aureus *and *M. catarrhalis *detected by PCR amongst PCV naive infants is described. Nine out of ten times, S. *pneumoniae *was co-carried with at least one other pathogen, most often *H. influenzae *and or *M. catarrhalis *(Figure 3). Multiple colonisation of the nasopharynx may have important clinical significance with regards to biofilm formation, polymicrobial infections and antibiotic resistance [28,29].

Here we report very early onset of colonisation similar to reports from high risk populations (Figure 1 and 2) [28,30]. 83% of the nasopharyngeal samples collected within a week of birth had between one and four pathogens detected (Figure 3) and the time to first acquisition was less than 8 weeks for of *S. pneumoniae, H. influenzae *and *M. catarrhalis*. In a culture-based study, Watson *et al*. [28] showed that by 6 months, 72%, 69% and 60% of rural Aboriginal infants from Western Australia had acquired *M. catarrhalis, S. pneumoniae and H. influenzae *respectively. In another culture-based study, *M. catarrhalis *colonization was reported to be as high as 100% amongst 60-day old Aboriginal infants [31].

This finding is comparable to data from the highlands of Papua New Guinea, by 3 months all infants had acquired *S. pneumoniae *[30]; whereas reports from other regions suggest much longer times to first acquisition for *S. pneumoniae*; 50% colonization by 8 weeks in Bangladesh [32], 34% colonization by 6 months in Finland [33] and average time to first detection of 6 months in the United States [14]. However, as these studies were culture-based, it is yet to be determined if the application of molecular tools would result in earlier detection of *S. pneumoniae *and the other pathogens.

Consistent with findings amongst Aboriginal and non-Aboriginal infants in Western Australia [28], *S. pneumoniae, H. influenzae *and *M. catarrhalis *point prevalence increased in the first few months of life. *S. pneumoniae *carriage exceeded 80% by 12 weeks, which was also reported in the larger cohort of 236 infants [11]. Although *S. aureus *had the highest point prevalence amongst the new-borns (> 50%) (Figure 1) and was acquired very rapidly by most infants (Figure 2), the prevalence declined to less than 20% by 9 weeks, similar to the report from Western Australian [28]. High nasopharyngeal carriage of *S. aureus *amongst the Gambian neonates may be linked with the high burden invasive *S. aureus *disease in this age group. *S. aureus *was the most frequently isolated pathogen amongst Gambian neonates (less than three months old) with serious infections excluding meningitis [34]. This finding has important implications for *S. aureus *vaccine development and scheduling.

The introduction of the Hib polysaccharide-tetanus toxoid conjugate vaccine in 1997 saw Hib carriage among Gambian children under five years drop from 12% to 0.25% (p < 0.01) [35-37]. In this study, Hib was found in 0.7% (95CI 0.5%, 0.9%) of the NP swabs and most *H. influenzae *detected was non-typeable (Figure 1). Low levels of carriage may explain why Hib is still responsible for a small proportion of IBD amongst Gambian infants despite widespread vaccination [26]. Low levels of Hib carriage in the Gambia may provide a "boosting" effect within the communities [38], preventing higher levels of re-emergence disease. Continued Hib vaccination as well as surveillance of Hib carriage and disease is of great importance in this region.

There is evidence of complex relationships between pneumococci and other respiratory pathogens that co-colonise the nasal and pharyngeal mucosae [39-41]. In this study, we found that *S. pneumoniae *had a positive association with *H. influenzae *and *M. catarrhalis *consistent with previous reports [28,42]. The association between *S. pneumoniae *and *H. influenzae *remained significant after adjusting for confounding factors. In contrast, the negative association between *S. pneumoniae *and *S. aureus *was reversed after adjusting for confounding factors. Although there is evidence of direct interference mechanisms between *S. aureus *and *S. pneumoniae *[43], host immune function may play an important role in modulating the association between these two pathogens [42].

The average prevalence of *S. pneumoniae *amongst the infants using *cpsA *gene-based PCR detection was 78% (95CI: 76%, 83%), very close to 79% (95CI: 75%, 82%) which was previously determined by culture for the same group of infants. *S. pneumoniae *was found in 36 (34%) of the 106 culture-negative samples, however, it was not detected in 34 (9%) of the 392 culture-positive samples [11]. The *cpsA *primers used do not detect all pneumococcal serotypes which could be accountable for the failed detections [44]. Broth-enrichment prior to molecular analysis has been shown to improve the molecular detection of low-density *S. pneumoniae *carriage [45]; however, it needs to be determined if broth-enrichment would give comparable enhancement for the other pathogens assayed. The detection of *S. pneumoniae *in the culture-negatives could be associated with the specificity of the *cpsA *gene primers used; hence, the inclusion of an alternate gene locus such as the autolysin gene (*lyt*A) which has been shown to have high specificity could be used to verify the data presented here [46]. The samples were stored at -70°C for up to five years prior to the molecular analysis. Kwambana *et. al*., provided preliminary evidence that deep frozen-storage of NP swabs in STGG may have differential effects on the detection of some bacterial taxonomic groups [47]. Hence, real time processing of nasopharyngeal swabs may be considered in future studies.

*S. aureus *had the lowest average prevalence among the infants (< 20%). The low rates of detection could be in part explained by the use of lysozyme for the extraction of nucleic acids. Lysozyme does not effectively degrade the *S. aureus *cell wall whereas the inclusion of lysostaphin has been shown to significantly improve the PCR-based detection of *S. aureus *[48]. An important limitation of using culture-independent molecular detection of pathogens is that important antibiotic resistance data cannot be determined.

## Conclusions

The results of this study suggest that *H. influenzae *and *M. catarrhalis *are ubiquitous inhabitants of the nasopharynx which are acquired early and frequently co-carried with *S. pneumoniae *in the Gambia. *S. aureus *prevalence is high among neonates, which may be associated with the high neonatal IBD burden in this region. This has important potential implications for the aetiology of respiratory polymicrobial infections, biofilm formation and vaccine strategies.

PCV-7 which markedly reduces carriage of vaccine and vaccine associated serotypes (> 60% of circulating pneumococci) in the Gambia is in widespread use [11]. A protein vaccine which may eliminate mucosal carriage of both *S. pneumoniae *and *H. influenzae *is currently under Phase 2 trials in the Gambia [49]. The effects of these vaccines on pharyngeal microbial carriage, particularly the pathogens that share the niche with *S. pneumoniae*, are yet to be described and warrant more attention.

## Competing interests

The authors declare that they have no competing interests.

## Authors' contributions

RAA, MA, and MRB established the framework for this study. BK, RAA and MA designed the study. BK prepared the clinical samples and performed all the experiments with MA. BK, CB and MA performed the data analysis. BK and MA wrote the manuscript with input from all. All authors contributed in discussions and approved the final manuscript.

## Pre-publication history

The pre-publication history for this paper can be accessed here:

http://www.biomedcentral.com/1471-2334/11/175/prepub
